# Alleviation of Hg-, Cr-, Cu-, and Zn-Induced Heavy Metals Stress by Exogenous Sodium Nitroprusside in Rice Plants

**DOI:** 10.3390/plants12061299

**Published:** 2023-03-13

**Authors:** Chrizostom Julius Niyoifasha, Birhanu Miressa Borena, Irasapa Tanimu Ukob, Phan Ngoc Minh, Tiba Nazar Ibrahim Al Azzawi, Muhammad Imran, Sajid Ali, Anousone Inthavong, Bong-Gyu Mun, In-Jung Lee, Murtaza Khan, Byung-Wook Yun

**Affiliations:** 1 Department of Applied Biosciences, Kyungpook National University, Daegu 41566, Republic of Korea; 2 Biosafety Division, National Institute of Agriculture Science, Rural Development Administration, Jeonju 55365, Republic of Korea; 3 Department of Horticulture and Life Science, Yeungnam University, Gyeongsan 38541, Republic of Korea

**Keywords:** sodium nitroprusside, radical oxygen species, gene expression, rice, heavy metals stress

## Abstract

The cultivation of rice is widespread worldwide, but its growth and productivity are hampered by heavy metals stress. However, sodium nitroprusside (SNP), a nitric oxide donor, has been found to be effective for imparting heavy metals stress tolerance to plants. Therefore, the current study evaluated the role of exogenously applied SNP in improving plant growth and development under Hg, Cr, Cu, and Zn stress. For this purpose, heavy metals stress was induced via the application of 1 mM mercury (Hg), chromium (Cr), copper (Cu), and zinc (Zn). To reverse the toxic effects of heavy metals stress, 0.1 mM SNP was administrated via the root zone. The results revealed that the said heavy metals significantly reduced the chlorophyll contents (SPAD), chlorophyll a and b, and protein contents. However, SNP treatment significantly reduced the toxic effects of the said heavy metals on chlorophyll (SPAD), chlorophyll a and b, and protein contents. In addition, the results also revealed that heavy metals significantly increased the production of superoxide anion (SOA), hydrogen peroxide (H_2_O_2_), malondialdehyde (MDA), and electrolyte leakage (EL). However, SNP administration significantly reduced the production of SOA, H_2_O_2_, MDA, and EL in response to the said heavy metals. Furthermore, to cope with the said heavy metals stress, SNP administration significantly enhanced the activities of superoxide dismutase (SOD), catalase (CAT), peroxidase (POD), and polyphenol peroxidase (PPO). Furthermore, in response to the said heavy metals, SNP application also upregulated the transcript accumulation of *OsPCS1*, *OsPCS2*, *OsMTP1*, *OsMTP5*, *OsMT-I-1a*, and *OsMT-I-1b*. Therefore, SNP can be used as a regulator to improve the heavy metals tolerance of rice in heavy-metals-affected areas.

## 1. Introduction

Increased anthropogenic activity, fast industrialization, and contemporary farming techniques over the past few decades have led to an increase in heavy metals poisoning in the environment, which is harmful to living organisms [1,2]. Both essential and nonessential heavy metals, including mercury (Hg), chromium (Cr), copper (Cu), and zinc (Zn), if present in excess, are lethal to plants [3,4]. The rising population, excessive use of fertilizers, inappropriate disposal of wastes, and increase in food consumption have made the situation much worse [1,2]. Heavy metals are reported to be detrimental to the health of humans, animals, plants, soil, and aquatic organisms [3].

The typical symptoms of essential and nonessential heavy metals in plants include the reduction in root and shoot lengths, the number of tillers per plant, chlorophyll and protein contents, and an increase in the production of reactive oxygen species (ROS), malondialdehyde (MDA), electrolyte leakage (EL), water and nutrients imbalances, chlorosis, and senescence, ultimately resulting in plant death [1,5,6]. When a cell is exposed to heavy metals, some cell organelles are impacted, including the cell membrane, nucleus, endoplasmic reticulum, mitochondria, lysosome, enzymes, and metabolites involved in the healing process after injury [7]. Due to their endurance in nature, heavy metals threaten human health and plants. For instance, Pb, one of the most hazardous heavy metals, is estimated to keep its high concentration for up to 150 years and has a soil retention duration of 150–5000 years [8]. Similarly, cadmium and Cr stress adversely affect the physical, biochemical, and molecular aspects of the plants [4,6]. The toxicity of heavy metals depends on several factors, including the dose, route of exposure, and chemical species, as well as the age, gender, genetics, and nutritional status of exposed individuals. Therefore, the removal of heavy metals ought to be prioritized in light of serious public health issues [9].

Plants have several defense systems against heavy metals stress and are in charge of preserving the homeostasis of the vital metals that plants need. Additionally, the focus of these systems is on shielding plants from exposure to soil-borne heavy metals or building plant tolerance by detoxicating the metals [10]. Other systems are more specialized and start when the appropriate stress is present. When exposed to the toxicity of heavy metals, a plant’s first line of defense consists of reducing the uptake of metals, and involves the assistance provided by cellular and root exudates, which prevent metals from entering the cell [10]. Many plants have specialized systems for different metal ions, and they work to compartmentalize these ions to prevent exposure to cells’ most delicate parts [10]. Other detoxification mechanisms for these metals are now in place, chelating, transporting, sequestering, and detoxifying these metal ions in the plant’s vacuole, serving as a second line of defense [5,10]. Furthermore, in response to heavy metals stress, plants produce hormones, phytochelatins (PCs), metal tolerance proteins (MTPs), metallothionein (MTs) ROS, reactive nitrogen species (RNS), and antioxidants [5].

Different techniques are used by plant scientists to improve the defense system of the plants against heavy metal stress, including the application of plant-growth-promoting rhizobacteria (PGPR), exogenous phytohormones and fertilizers, melatonin (MT), and donors of nitric oxide (NO) [5,11,12,13,14]. As a signaling molecule, NO significantly contributes to regulate plant growth and development and defense responses against biotic and abiotic stress conditions [15,16,17,18,19]. After 1 mM S-nitrosocysteine (CySNO) infiltration into *Arabidopsis thaliana*, 6436 genes (3448 upregulated and 2988 downregulated) and 6214 transcripts (3335 upregulated and 2879 downregulated) displayed differential expression [20]. These differentially expressed genes were discovered to be involved in important physiological activities, such as hormone signaling, other developmental processes, and plant defense against various biotic and abiotic stressors [18,20]. Recent studies showed that applying SNP improved the ability of the rice, soybean, and Mexican lime plants to withstand lead, flooding, salt, and drought stress, respectively [5,21,22,23]. Therefore, considering the multifaceted role of NO under abiotic stress conditions in different plant species, the present study was conducted to assess the mitigating effects of SNP on Hg, Cr, Cu, and Zn stress in the Korean rice cultivar Jinbu (*Oryza sativa* L.).

## 2. Materials and Methods

### 2.1. Plant Materials and Growth Conditions

The experiment was conducted in a greenhouse at Kyungpook National University in Daegu, Republic of Korea, under controlled conditions with a temperature of 25 ± 1 °C, a 16 h light–dark cycle, and a 60 ± 2% humidity level. The Jinbu rice cultivar was used in the current study. The previously mentioned techniques were applied for seed sterilization, germination, sowing, and application of Hg, Cr, Cu, Zn, and SNP through the root zone [2,5]. To induce heavy metals stress, mercury, chromium (VI) oxide (Sigma-Aldrich, St. Louis, MO, USA), cadmium sulfate hydrate, and zinc nitrate hexahydrate were used as donors. One mM of each heavy metal was applied to the soil (Doobeana Plus, Nong Kyung Ltd., Yeongcheon-si, Republic of Korea) four weeks before transplantation. Until the time of the transplant, the treatment was repeated every three days. Then, 4-week-old rice seedlings that were healthy, active, and uniform in height were transferred to 21 × 15 cm pots filled with heavy-metals-contaminated soil. A total of ten pots were used per treatment, and each pot was transplanted with four seedlings. After three weeks of transplantation, the plants were supplied with 0.1 mM SNP [5]. The treatments were as follows: (1) control (only water), (2) 0.1 mM SNP, (3) 1 mM Hg, (4) 1 mM Cr, (5) 1 mM Cu, (6) 1 mM Zn, (7) 1 mM Hg + 0.1 mM SNP, (8) 1 mM Cr + 0.1 mM SNP, (9) 1 mM Cu + 0.1 mM SNP, and (10) 1 mM Zn + 0.1 mM SNP.

### 2.2. Sample Collection and Phenotypic Evaluation under Heavy Metals Stress

Data were recorded for chlorophyll content (soil and plant analysis development (SPAD) values) and electrolyte leakage (EL). In addition, samples were collected for chlorophyll a and b, protein, superoxide anion (SOA), malondialdehyde (MDA), hydrogen peroxide (H_2_O_2_), antioxidant, and transcript accumulation analyses [2,5].

### 2.3. Measurement of Chlorophyll and Protein Contents

An SPAD meter was used to measure the leaves’ chlorophyll concentration (SPAD-502, Minolta Co., Ltd., Osaka, Japan). Furthermore, chlorophyll a and b contents were measured as described earlier [24]. Briefly stated, 0.5 g of fresh plant material was homogenized in 80% acetone, vortexed for 2 min, and then incubated for 30 min at room temperature. After centrifuging the supernatant at 11,000× *g* for 10 min at 4 °C, the absorbance of the supernatant was measured at 470, 645, and 663 nm. The chlorophyll contents were measured as previously described [25]. The amount of soluble protein was measured as previously described [26]. Briefly, 10 mL of phosphate buffer (pH 7.0) was used to homogenize 500 mg of fresh plant material. This mixture was then vortexed and centrifuged at 10,000× *g* for 20 min at 4 °C. The extract was mixed with SERVA Blue-G (Universal Biologicals Ltd., Cambridge, UK); the absorbance was measured at 570 nm, and the soluble protein content was determined using a standard curve (mg/g FW). The results were calculated using the following formula:

(C×Vt)=(W×Vs×100)

where C is the protein content, Vt is the total volume of the reaction mixture, Vs is the volume of the supernatant, and W is the weight of the fresh plant sample.

### 2.4. Measurement of SOA, H_2_O_2_, MDA, and EL

The Imran et al. [27] approach was applied to determine SOA. In brief, 0.2 g of fresh leaves were homogenized in 2 mL of phosphate buffer (50 mM, pH 7.8) before being centrifuged at 10,000× *g* for 15 min at 4 °C. The mixture was then incubated at room temperature (RT) for 25 min with 0.1 mL of 10 mM hydroxylamine hydrochloride and 0.5 mL of phosphate buffer (50 mM, pH 7.8). After incubation, 1 mL of 7 mM naphthylamine and 1 mL of 17 mM sulfanilamide were added to the mixture and further incubated at room temperature for 25 min. At 530 nm, the absorbance was measured, and, using a NaNO_2_ standard curve, the SOA production was determined (expressed as µmol/g FW).

A previously published technique was used to measure the H_2_O_2_ content [28]. In brief, 5 mL of 0.1% trichloroacetic acid (TCA) was used to extract 0.2 g of the leaf sample, which was then centrifuged at 12,000× *g* for 15 min at 4 °C. The absorbance at 390 nm was measured after the supernatant (0.5 mL) was collected, then 1 mL of 1 M potassium iodide, and 0.5 mL of 10 mM phosphate buffer (pH 7.0) were added. The H_2_O_2_ content (expressed as µmol/g FW) was estimated using the extinction coefficient (ε) = 0.28 mM/cm [27].

Based on MDA values, as previously indicated, lipid peroxidation in the leaves was calculated [25]. Simply stated, 0.1 g of fresh plant tissue was combined with 10 mL of 5% TCA and centrifuged for 10 min at 6000× *g* at 4 °C. After being heated for 25 min at 90 °C and immediately cooled to 4 °C, the supernatant was suspended in 4 mL of tribromoacetic acid and centrifuged at 6000× *g* for 10 min at 4 °C. At 532 and 600 nm, the absorbance of the supernatant was measured.

Furthermore, we evaluated ion leakage brought on by heavy-metals-related oxidative damage using an EL assay damage [2]. In order to remove surface electrolytes, about 200 mg of fresh leaf samples from the heavy-metals-treated and control plants were rinsed with deionized water. The samples were put in test tubes with 10 mL of deionized water and kept at room temperature for 6 h. To detect electrolyte leakage 1 (EL1; step 1), a transportable conductivity meter (HURIBA Twin Cond B-173, Japan) was used. In order to determine electrolyte leakage 2 (EL2; step 2), leaf samples from step 1 were autoclaved at 120 °C for 15 min. Ion leakage was computed as the proportion of steps 1 and 2 and given as the ratio of EL1/EL2 × 100.

### 2.5. Antioxidant Enzymatic Assay

The photochemical reduction of nitro blue tetrazolium (NBT) was used to evaluate SOD activity as previously described [27,29]. SOD activity units were calculated as the enzyme concentration needed to block NBT degradation by 50% when measured at 560 nm.

As previously mentioned [27], the H_2_O_2_ absorbance reduction at 240 nm was calculated to measure the CAT activity. The reaction buffer included 15 mM H_2_O_2_ and 50 mM potassium phosphate buffer (pH 7.8). Then, to start the reaction, 100 µL of the enzyme extract was added to the reaction mixture. Utilizing an extinction coefficient (ε) of 40 mM/cm, the H_2_O_2_ concentration in the reaction mixture was measured to determine the CAT activity after 1 min.

POD activity was measured using the guaiacol method [30], which involved adding 0.1 mL of the reaction mixture made up of 1.0 mL of 2% H_2_O_2_, 2.9 mL of 50 mM phosphate buffer (pH 5.5), and 1.0 mL of 50 mM guaiacol to the supernatant. As a control, phosphate buffer without enzyme was employed. POD activity was determined as the unit change per minute after reading the absorbance at 470 nm for 3 min [27].

PPO activities were assessed using the methodology developed by [2]. The assay reaction mixture for PPO contained 50 µL of crude enzyme extract, 100 µL of phosphate buffer (0.1 M), and 50 µL of pyrogallol (50 µM). A wavelength of 420 nm was used to measure the absorbance. The computations were performed as previously described [31].

### 2.6. Quantitative Real-Time PCR (qRT-PCR)

Following the manufacturer’s instructions, total RNA from the leaves was extracted using the TRIzol reagent. Then, complementary DNA (cDNA) was synthesized from RNA by using a BioFACT RT kit. Gene expression analysis was performed using the synthesized cDNA as a template. Using the Eco™ real-time PCR system, a 20 µL reaction mixture containing 2X Real-Time PCR Master Mix [(including SYBR^®^ Green I) BIOFACT, Korea] and 10 nM of each primer was processed (Illumina, USA). The negative control was a no-template control (NTC) using nuclease-free water instead of the cDNA template. Initial denaturation took place at 95 °C for 15 min, then there were 40 cycles of 95 °C for 10 s and 60 °C for 30 s. The internal reference gene was Actin [18]. The expression of heavy-metals-stress-related genes, including *OsPC1*, *OsPC2*, *OsMTP1*, *OsMTP5*, *OsMT-1-1a*, and *OsMT-1-1b*, was analyzed. Supplementary Table S1 lists the genes and related primers along with their names and sequences.

### 2.7. Statistical Analysis

Each experiment was repeated thrice, and all treatments were replicated at least thrice. Means were derived using data of all replicates from all experiments. Using Statistical Analysis Software (SAS, version 9.1), mean values were compared by using Duncan’s multiple range test (DMRT) at a significance threshold (*p* < 0.05). Statistical Analysis System (SAS 9.1) was used for DMRT analysis to evaluate the significance of each treatment. GraphPad Prism (version 6.0, GraphPad, San Diego, CA, USA) was used to visualize the data.

## 3. Results

### 3.1. SNP Application Improved Plant Vigor, Chlorophyll (SPAD), Chlorophyll a and b, and Protein Contents

**Figure 1:**
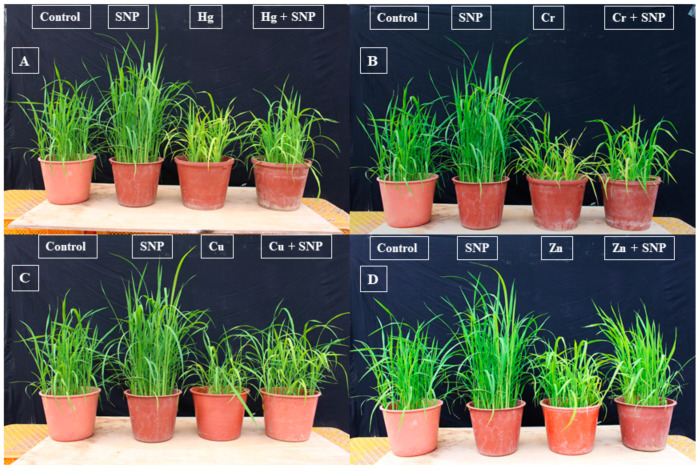
Effects of exogenously applied SNP on rice vigor with or without Hg-, Cr-, Cu-, and Zn-induced stress. (**A**) Control, SNP, Hg, Hg + SNP, (**B**) Control, SNP, Cr, Cr + SNP, (**C**) Control, SNP, Cu, Cu + SNP, (**D**) Control, SNP, Zn, Zn + SNP.

**Figure 2:**
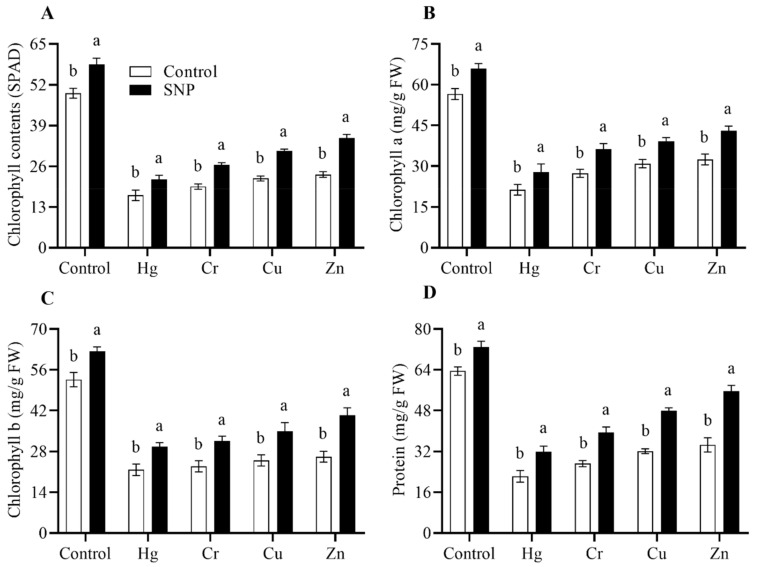
Effects of exogenously applied SNP on rice with or without Hg-, Cr-, Cu-, and Zn-induced stress on rice plants. (**A**) Chlorophyll (SPAD), (**B**) chlorophyll a content, (**C**) chlorophyll b content, and (**D**) protein content. Each data point indicates the mean ± standard deviation (*n* = 3). Bars with different letters indicate significant differences, according to Duncan’s multiple range test. The results are compared to the respective controls (Hg-, Cr-, Cu-, and Zn-untreated and Hg-, Cr-, Cu-, and Zn-treated plants).

The phenotypic evaluation revealed that the application of SNP significantly improved plant vigor under normal and Hg-, Cr-, Cu-, and Zn-induced heavy metals stress conditions (Figure 1). The results also revealed that SNP application increased chlorophyll (SPAD) (16%), chlorophyll a (19%), b (13%), and protein (9%) contents more than control plants (only treated with water) (Figure 2). Exposure to Hg, Cr, Cu, and Zn significantly reduced chlorophyll (SPAD) contents by 61, 55, 49, and 39%, respectively, as compared to control plants (only treated with heavy metals) (Figure 2). Similarly, the said heavy metals significantly reduced chlorophyll a and b contents by (52 and 51%), (48 and 49%), (42 and 44%), (39 and 41%), respectively, as compared to control plants (only treated with water) (Figure 2). In a similar pattern, the said heavy metals also significantly reduced the protein contents by 62, 53, 45, and 41%, respectively, as compared to control plants (only treated with water) (Figure 2). However, SNP application significantly reduced the toxic effects of the said heavy metals on chlorophyll (SPAD), chlorophyll a and b, and protein contents by (30, 32, 36 and 43%), (35, 35, 38 and 45%), (40, 39, 40 and 49%), and (50, 46, 54 and 55%), respectively, as compared to control plants (only treated heavy metals) (Figure 2).

### 3.2. In Response to Heavy Metals Stress, SNP Application Significantly Reduced the Production of SOA, H_2_O_2_, MDA, and EL

**Figure 3:**
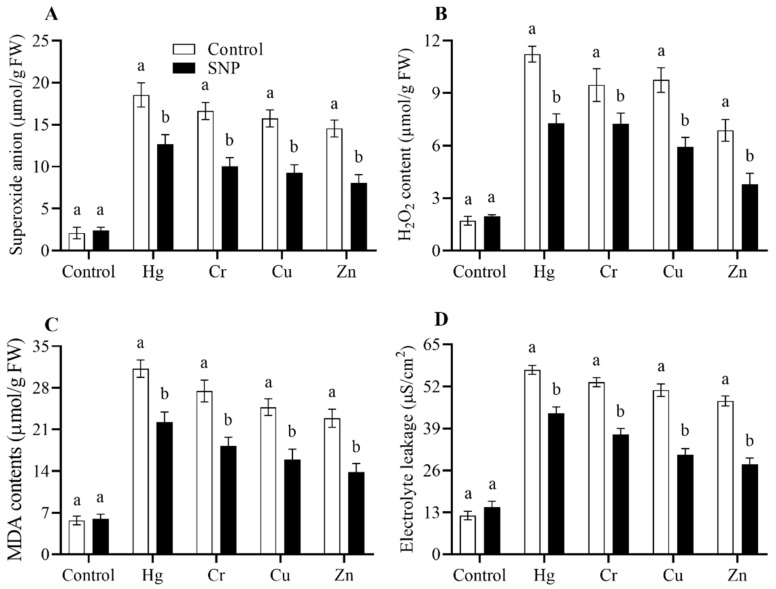
Effects of exogenously applied SNP on rice with or without Hg-, Cr-, Cu-, and Zn-induced stress on rice plants. (**A**) Superoxide anion level, (**B**) H_2_O_2_ content, (**C**) MDA level, (**D**) electrolyte leakage. Each data point indicates the mean ± standard deviation (*n* = 3). Bars with different letters indicate significant differences, according to Duncan’s multiple range test. The results are compared to the respective controls (Hg-, Cr-, Cu-, and Zn-untreated and Hg-, Cr-, Cu-, and Zn-treated plants).

In the present investigation, we evaluated the effects of heavy metals stress on the production of ROS, including SOA, H_2_O_2_, MDA, and EL, and the role of SNP in the reduction of these molecules to reduce their toxic effects. The results from the current study showed that the application of heavy metals (Hg, Cr, Cu, and Zn) significantly increased the production of SOA, H_2_O_2_, MDA, and EL (Figure 3). However, the treatment of SNP significantly reduced the production of SOA, H_2_O_2_, MDA, and EL induced by Hg, Cr, Cu, and Zn by (32, 40, 41, and 44%), (30, 40, 39, and 45%), (29, 33, 36, and 40%), and (23, 30, 39, and 41%), respectively, as shown in Figure 3.

### 3.3. SNP Application Regulates the Antioxidant System

**Figure 4:**
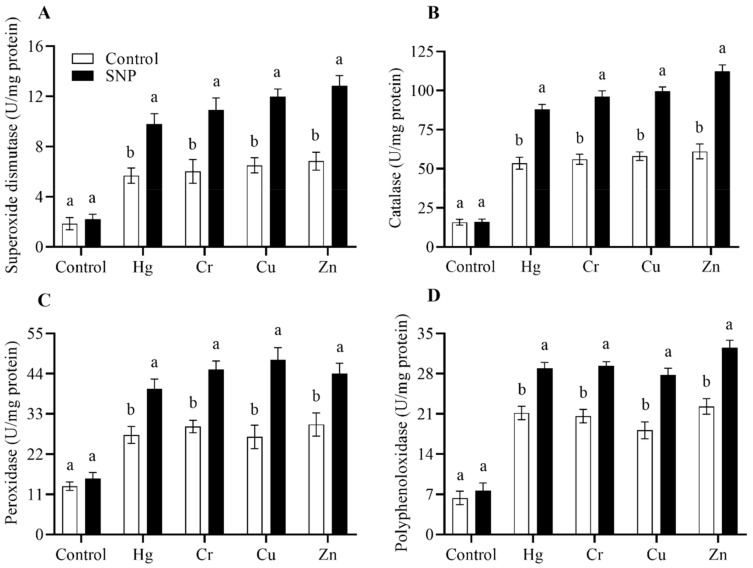
Effects of exogenously applied SNP on rice with or without Hg-, Cr-, Cu-, and Zn-induced stress on rice plants. (**A**) Superoxide dismutase, (**B**) catalase, (**C**) peroxidase, (**D**) polyphenol oxidase. Each data point indicates the mean ± standard deviation (*n* = 3). Bars with different letters indicate significant differences, according to Duncan’s multiple range test. The results are compared to the respective controls (Hg-, Cr-, Cu-, and Zn-untreated and Hg-, Cr-, Cu-, and Zn-treated plants).

In response to heavy metals stress, plants overproduce ROS, which cause oxidative damage. Plants inhibit the synthesis of ROS by triggering the antioxidants SOD, CAT, POD, and PPO. We also found that in response to heavy metals stress (Hg, Cr, Cu, and Zn), SNP application significantly increased the activities of these antioxidants by (72, 81, 84, and 88%), (64, 71, 78, and 83%), and (46, 53, 57, and 60%), respectively, as shown in Figure 4.

### 3.4. SNP Application Alters the Expression of Heavy-Metals-Stress-Related Genes

**Figure 5:**
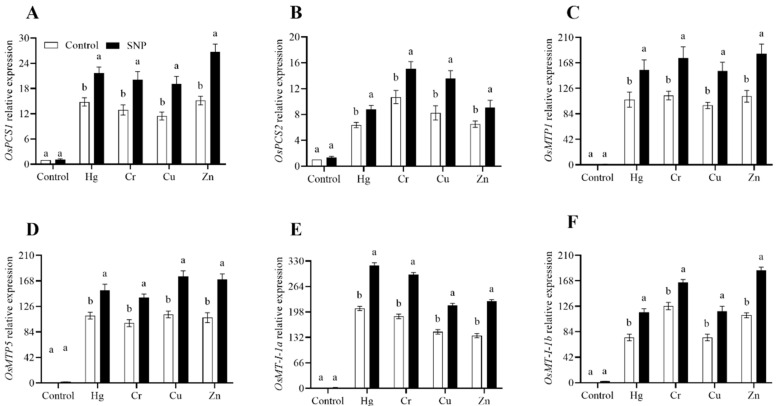
Effects of exogenously applied SNP on rice with or without Hg-, Cr-, Cu-, and Zn-induced stress on rice plants. (**A**) *OsPC1*, (**B**) *OsPC2*, (**C**) *OsMTP1*, (**D**) *OsMTP5*, (**E**) *OsM1-1a*, *(***F**) *OsM1-1b*. Each data point indicates the mean ± standard deviation (*n* = 3). Bars with different letters indicate significant differences, according to Duncan’s multiple range test. The results are compared to the respective controls (Hg-, Cr-, Cu-, and Zn-untreated and Hg-, Cr-, Cu-, and Zn-treated plants).

To decrease the toxic effects of heavy metals stress, plants dramatically boost the expression of genes related to this stress. The results from the current study revealed that in response to Hg, Cr, Cu, and Zn, SNP treatment significantly increased the expression of *OsPCS1*, *OsPCS2*, *OsMTP1*, *OsMTP5*, *OsMT-I-1a*, and *OsMT-I-1b* by (47, 55, 67, and 76%), (38, 41, 65, and 40%), (45, 54, 58, and 62%), (38, 43, 55, and 59%), (54, 58, 47, and 65%), and (56, 31, 58, and 66%), respectively, as shown in Figure 5.

## 4. Discussion

Plants exposed to heavy metals stress experience profound physiological, molecular, and biochemical changes [2,3,6]. However, the application of PGPRB, melatonin, exogenous phytohormones and fertilizers, and different NO donors significantly reduced the toxic effects of abiotic stresses, including heavy metals stress [5,11,14,22,32]. In the current study, similar results were also observed. Exposure to Hg, Cr, Cu, and Zn stress negatively affected the vigor of rice plants (Figure 1) and dramatically decreased the chlorophyll (SPAD), chlorophyll (a and b), and protein contents (Figure 2). However, SNP application significantly reduced the toxic effects of Hg, Cr, Cu, and Zn on the chlorophyll (SPAD) content of rice plants by 61, 55, 49, and 39%, respectively, as compared to control plants (only treated with heavy metals) (Figure 2). In addition, compared to control plants (only treated with water), the aforementioned heavy metals considerably decreased the amounts of chlorophyll a and b by 52 and 51%, 48 and 49%, 42 and 44%, and 39 and 41%, respectively (Figure 2). In a similar vein, the aforementioned heavy metals drastically decreased the protein contents of the rice plants by 62, 53, 45, and 41%, respectively, in comparison to the control plants (only treated with water) (Figure 2). Emamverdian et al. [33] and Rahim and Khan et al. [5] also noticed similar results, and they suggested that SNP application significantly reduced the toxic effects of lead and cadmium stress on bamboo and rice plants by increasing their chlorophyll and protein contents.

According to Khan et al. [2,3], when rice plants are stressed by heavy metals, a significant number of ROS are produced, which results in oxidative damage. However, NO serves as an ROS scavenger to shield rice plants from their hazardous effects [5,34]. In the current experiment, comparable outcomes were also seen. The generation of ROS was significantly elevated when rice plants were exposed to Hg, Cr, Cu, and Zn stress. However, SNP treatment considerably decreased the production of SOA, H_2_O_2_, and MDA and EL by 32, 40, 41, and 44%, 30, 34, 39, and 45%, 29, 33, 36, and 40%, and 23, 30, 39, and 41%, respectively, as shown in Figure 3. In response to Cu- and Pb-induced oxidative stress on rice, SNP application significantly reduced the production of ROS through the activation of the antioxidant system [5,34].

Plants reduce the overproduced ROS by the activation of the antioxidant system. NO as a signaling molecule increases the activities of antioxidants including SOD, CAT, POD, and PPO [35,36]. Our results also revealed that in response to Hg, Cr, Cu, and Zn stress, SNP application boosted the production of these antioxidants by 72.27, 81, 84, and 88%, 64, 71, 78, and 83%, and 46, 53, 57, and 60%, respectively, as shown in Figure 4.

To cope with heavy metals stress conditions, including Cd, Pb, and arsenic, plants increase the expression of *OsPCS1*, *OsPCS2*, *OsMTP1*, *OsMTP5*, *OsMT-I-1a*, and *OsMT-I-1b* [5,35,37]. We were also interested in how these genes responded to Hg, Cr, Cu, and Zn stress and how they would advance with the addition of SNP. We also found that in response to heavy metals stress, SNP application significantly increased the transcript accumulation of these genes by 47, 55, 67, and 76%, 38, 41, 65, and 40%, 45, 54, 58, and 62%, 38, 43, 55, and 59%, 54, 58, 47, and 65%, and 56, 31, 58, and 66%, respectively, as shown in Figure 5. Rahim and Khan et al. [22] also observed that the application of different NO donors increased the expression of abiotic-stress-related genes including *OsPCS1*, *OsPCS2*, *OsMTP1*, *OsMTP5*, *OsMT-I-1a*, and *OsMT-I-1b*. Overall, the current results revealed that SNP administration can be used as a regulator to improve the heavy metals stress tolerance of rice in heavy-metals-affected areas. However, the application of SNP on a massive scale requires detailed studies and economic justifications. Various studies have reported the positive effects of SNP in the alleviation of abiotic stressors; however, researchers should turn their attention to investigating the unwanted effects of SNP on crops and soil.

## 5. Conclusions

The growth and development of rice plants were adversely affected under heavy metals stress. However, SNP application significantly enhanced chlorophyll (SPAD), chlorophyll (a and b), and protein contents and dramatically reduced the production of ROS (SOA, H_2_O_2_, and MDA) and EL. In addition, SNP application reduced the oxidative damages caused by ROS by increasing the activities of antioxidants, including SOD, CAT, POD, and PPO. The defense system of the rice plants against heavy metals stress was further improved by SNP via the induction of *OsPCS1*, *OsPCS2*, *OsMTP1*, *OsMTP5*, *OsMT-I-1a*, and *OsMT-I-1b* genes. Based on the present results, it is suggested that the application of SNP could enhance the growth and productivity of rice plants under heavy metals stress. Moreover, the role of SNP in the mitigation of heavy metals stress revealed higher efficiency in the case of Zn compared to Hg, which needs to be further investigated. Similarly, further studies are required to optimize the dose, investigate expressions of other candidate genes, impact on crops yields, and expenses of SNP for sustainable agriculture under heavy metals stress.

## Data Availability

The datasets generated and/or analyzed during the current study are available from the corresponding authors upon reasonable request.

## Supplementary Materials

The following supporting information can be downloaded at: https://www.mdpi.com/article/10.3390/plants12061299/s1, Table S1: List of primers used in this study.
