# Supplementary material for: Alleviation of Hg-, Cr-, Cu-, and Zn-Induced Heavy Metals Stress by Exogenous Sodium Nitroprusside in Rice Plants
Source: Plants (Basel). 2023 Mar 13;12(6):1299. doi: 10.3390/plants12061299 (PMC10056095; doi:10.3390/plants12061299)
Supplement: Supplementary file 1 [file plants-12-01299-s001.zip › plants-2235968-supplementary.pdf]

Table S1. List of primers used in this study.

Primer	Forward Sequence (5`-3`)	Reverse Sequence (5`-3`)
OsActin	GGA ACT GGT ATG GTC AAG GC	AGT CTC ATG GAT AAC CGC AG
OsPCS1	CGA AGA TTC CAT TTC CCA GA	TCG AGG ATA TCG GTG AAA GC
OsPCS2	TCC CTC TCC GTC GTC CTC	CCT CCG CCT TCA CCT TGT
OsMTP1	TCA AGA TGC TGC GCA ACA TCC	GAG CTC CTA CTC GCG CTC AAT G
OsMTP5	ACG CTC GTT GTC TGA TGG G	GTC ACT GCA AGC ATG ATG TCC AC
OsMT-I-1a	TGC GGA AG TAC CCT GA	TTC TCC GGC GCC ACA C
OsMT-I-1b	CTG TGG ATC AAG CTG TGG CT	GCT GCT GCT CTT CTC TTC CA
